# *In Vitro* Gastrointestinal Digestion of Corn-Oil-in-Water Pickering Emulsions: Influence of Lignin-Containing Cellulose Nanofibrils Loading

**DOI:** 10.3390/polym16182648

**Published:** 2024-09-19

**Authors:** Langhong Wang, Lin Liu, Jun Li, Jianming Liao, Bin Li, Wenjuan Jiao, Shasha Guo

**Affiliations:** 1 Guangdong Provincial Key Laboratory of Intelligent Food Manufacturing, College of Food Science and Engineering, Foshan University, Foshan 528225, China; wlhong@fosu.edu.cn; 2 Sericulture & Agri-Food Research Institute Guangdong Academy of Agricultural Sciences, Key Laboratory of Functional Foods, Ministry of Agriculture and Rural Affairs, Guangdong Key Laboratory of Agricultural Products Processing, Guangzhou 510610, China; shimlyliulin@163.com; 3 State Key Laboratory of Pulp and Paper Engineering, South China University of Technology, Guangzhou 510640, China; ppjunli@scut.edu.cn (J.L.); liaojm@qzc.edu.cn (J.L.); 4 College of Chemical and Material Engineering, Quzhou University, Quzhou 324000, China; 5 CAS Key Laboratory of Biofuels, Qingdao Institute of Bioenergy and Bioprocess Technology, Chinese Academy of Sciences, Qingdao 266101, China; libin@qibebt.ac.cn

**Keywords:** lignin-containing cellulose nanofibrils, Pickering emulsions, lipid digestion, free fatty acid release, gastrointestinal tract digestion

## Abstract

There is a growing trend in incorporating biomass-based engineered nanomaterials into food products to enhance their quality and functionality. The zeta potential, droplet size, microstructure, and content of free fatty acid (FFA) release were determined to investigate the influence of a plant-derived particle stabilizer, i.e., lignin-containing cellulose nanofibrils (LCNFs). Remarkable differences were observed during digestion stages, which were found to be correlated with the concentrations of LCNFs. The gradual FFA release in the small intestine stage from LCNF-coated lipid droplets was monitored over time, with a final lowest release of FFAs amounting to 26.3% in the emulsion containing 20.0% (*v*/*v*) of the dispersed phase stabilized by 3 mg/mL of LCNFs. This release can be attributed to the physical barrier at lipid droplet surfaces and the network effect created by the free LCNFs in the continuous phase. This work provides a foundation for the potential application of nature-derived LCNF materials in reducing fat absorbance.

## 1. Introduction

Overweight and obesity are prominent risk factors for numerous noncommunicable diseases, such as cardiovascular diseases, diabetes, and digestive disorders, which caused an estimated 5 million deaths in 2019 [1,2]. Numerous factors leading to overweight and obesity can be prevented and reversed, for instance by decreasing the intake of calories from fats and sugars. The primary approach to designing low-calorie alternatives is to use fat substitutes instead of fat globules, such as nanocellulose, whey protein microgels, polysaccharide colloids, olestra, and whey protein microgels [3,4]. However, decreasing the content of added fats can frequently influence the quality of food items [5]. Therefore, effective and innovative methods are required to maintain the texture of fats and delay lipid digestion, facilitating their seamless integration into daily dietary practices.

Recently, there has been a growing interest in fabricating a delivery system that will promote the delivery of undigested lipids to the ileum, aiming to enhance satiety and reduce calorie intake [6]. Digestion behavior can be hindered by coating different kinds of emulsifiers, such as small-molecule surfactants [7], proteins [8], nanochitin [9], or nanocellulose [10]. Compared with traditional emulsions, Pickering emulsions stabilized by colloidal particles are more effective in inhibiting lipid digestion during digestion. This phenomenon is due to the stronger resistance to droplet coalescence and higher desorption energy of Pickering stabilizers [11,12]. The high desorption energy of particle stabilizers poses a challenge for bile salts to displace them from the lipid surface, thereby hindering the subsequent lipase adsorption [13]. The selection of colloidal particles for formulating food-grade Pickering emulsions requires careful consideration of their weakness and strength to ensure optimal lipid digestibility and bioavailability performance. For example, protein-based particles are sensitive to highly acidic conditions and susceptible to pepsin-induced hydrolysis in the stomach, resulting in the exposure of lipids to the lipase in the small intestine [14]. Fabricating Pickering emulsions using a more stable and robust particle stabilizer is an excellent approach to prolonging lipid hydrolysis. This, in turn, can increase the quantity of undigested lipids reaching the ileum, thereby enhancing satiety.

Cellulose, a dietary fiber primarily sourced from plant cell walls, constitutes lignocellulose, which is the most abundant, renewable, and cost-effective natural resource [15,16]. Previous research has highlighted nanocellulose colloidal particles as ideal candidates for stabilizing Pickering emulsions due to their stronger resistance to droplet coalescence and higher desorption energy [17,18,19]. Their high desorption energy can inhibit the subsequent lipase adsorption and activity [20]. Lignin-containing cellulose nanofibrils (LCNFs) are more eco-friendly than their alternatives [21,22]. Retaining lignin in nanocellulose as a non-modifying surface and interface engineering strategy offers numerous advantages. Lignin, a native component, has the capability to modify the surface properties of nanocellulose, thereby impacting the interfacial characteristic of Pickering emulsions [23,24,25]. For example, this combination can improve the water resistance capability of cellulose materials [26] and also displays excellent mechanical properties independent of environmental moisture [27]. Nevertheless, limited research has been dedicated to exploring how LCNFs can effectively create a digestible physical barrier around oil droplets and their suitability for regulating lipid digestion under gastrointestinal conditions.

Following our previous research that focused on improving interfacial stabilization efficiency by controlling the lignin content in LCNFs, we aim to determine the digestion behavior of these emulsions. This study examined variations in the droplet diameter, zeta potential, and microstructure during different phases of digestion, along with free fatty acid (FFA) levels, to elucidate the underlying mechanism. The findings of this study will broaden the utilization of nature-derived nanocellulose materials in functional foods, with the goal of reducing fat absorption for weight management.

## 2. Materials and Methods

### 2.1. Materials

*p*-TsOH, styrene (purity 98.0%), and 2,2-azobisisobutyronitrile (AIBN) were purchased from Shanghai RichJoint Chemical Reagents (Shanghai, China), Damao Chemicals (Tianjin, China), and Fuchen Chemicals (Tianjin, China), respectively. Corn oil was purchased from COFCO Inc. (Shanghai, China). Nile red, Mucin (M2378), pepsin (P6887), bile extract (48305), sodium caseinate (C8654), and porcine pancreatin (P7545) were obtained from Sigma-Aldrich Chemicals (Shanghai, China). All the other reagents were analytical-grade chemicals.

### 2.2. Preparation of LCNFs

Eucalyptus wood chips were used as raw materials to produce unbleached mechanical pulp, with the primary components being approximately 51.0 wt% glucose, 14.0 wt% xylose, 0.3 wt% galactose, and 27.0 wt% lignin. Briefly, the washed wood chips were hydrothermally treated using a horizontal rotary digester (2611, Kumagai Riki Kogyo Co., Ltd., Tokyo, Japan) at 165 °C for 2.5 h. Afterward, extraction was conducted using an ethanol/toluene solvent (*v*/*v* = 1/2). Finally, the extraction process was carried out through continuous high-consistency refining (2500-II, Kumagai RikiKogyo Co., Ltd., Tokyo, Japan) with a disk gap of ~0.1 mm.

The preparation of LCNFs from pulp was according to our published report [28]. First, 10 g of pulp was added to a 75.0 wt% *p*-TsOH solution with liquid-to-solid ratio of 20:1 L/kg. Then, the suspension was operated at 200 rpm and 65 °C for 30 min with a mechanical mixer. After vacuum filtration cleaning, the suspension was homogenized using a microfluidizer (Mini, Noozle Fluid Technology Co., Ltd., Shanghai, China) to produce LCNFs. The LCNF suspension was kept at 4 °C and used within three months.

### 2.3. LCNF Characterization

Morphology. The microstructure of the LCNFs was assessed using a transmission electron microscope (TEM, JEM-1400 Plus, JEOL Ltd., Akishima-shi, Japan) and an atomic force microscope (AFM, MultiMode 8, Bruker Inc., Karlsruhe, Germany). For the AFM observation, the LCNF suspension was placed on cleaved mica and dried at room temperature. Subsequently, high-resolution AFM images were acquired in ambient air using tapping mode. The height of the LCNFs was analyzed using NanoScope Analysis 1.5 software (Bruker).

TEM was also used to acquire images of aggregated LCNFs at 80 kV. First, 5 µL of the nanofibril suspension (0.2 wt%) was placed on the grid (30 s) and then adsorbed by paper. Next, a 5 µL uranyl acetate solution (2.0%) was used for 30 s for negative staining; then, the excess liquid was removed by blotting with filter paper and dried at ambient temperature.

Interfacial tension. The corn oil–water interfacial tension was monitored using a Wilhelmy plate (DataPhysics DCAT 11, DataPhysicsInstruments GmbH, Filderstadt, Germany) in the presence of LCNFs. Each sample was tested in triplicate.

### 2.4. Preparation of Pickering Emulsions

The preparation of Pickering emulsions was conducted using a previous method with slight modifications [28]. The coarse emulsion was mixed with 20.0 wt% of corn oil and 80.0 wt% of the LCNF solution using a high-speed shear emulsifying machine (T18, IKA, Staufen, Germany) at 10,000 rpm for 30 s. Then, the coarse emulsion was further emulsified by an ultrasonic device (VCX800, SONICS, Connecticut, USA) with 30.0% power for 40 s (3 s sonication, 3 s interval) to create a fine emulsion. The concentrations of the LCNF solution were 1, 2, and 3 mg/mL, respectively, which were referred to as initial samples for digestion assays. To compare the degree of digestion, another emulsion was prepared using sodium caseinate as an emulsifier at a concentration of 0.3 wt%. The emulsion was prepared following the process of LCNF-stabilized Pickering emulsions.

To examine the surface morphology, the Pickering emulsion was prepared with styrene (St) and LCNF suspensions. Polystyrene (PS) beads were formed by free-radical polymerization of styrene monomers in the styrene phase under the action of the initiator AIBN [29]. The oil phase consisted of St and oil-soluble AIBN at a 1/100 (*w*/*w*) ratio. Afterwards, the St/AIBN was mixed with the LCNF suspension at a 2/8 (*w*/*w*) ratio. The preparation of the emulsion was same as the corn oil emulsions. After degassing for 30 min using nitrogen gas, polymerization of the emulsion was initiated at 65 °C, and the polymerization was continued for 18 h at 65 °C with stirring.

### 2.5. Simulated Gastrointestinal Tract Digestion Model and Free Fatty Acid Release

The Simulated Gastrointestinal Tract Digestion Model was performed using the INFOGEST digestion simulation method [30]. A test sample with a lower oil concentration (5.0 wt%) was obtained by appropriate dilution.

Oral phase: the test sample was added to simulated saliva fluid with mucin (0.00375 g/mL) and CaCl_2_•2H_2_O (1.5 mM). After the pH adjustment to 7.0, the mixture was kept at 37 °C for 2 min in a water bath (SHA-B, Lichen Technology, Shanghai, China) at 100 rpm, forming an oral bolus. Gastric phase: simulated gastric fluid (SGF) containing pepsin (2000 U/mL) was added to the ‘‘bolus’’ at a 1:1 volume ratio. The system was kept at 37 °C for 2 h after the pH adjustment to 3.0. Small intestinal phase: the “chime” after the gastric digestion was then diluted 1:1 (*v*/*v*) with simulated intestine fluid (SIF) containing 10 mM bile salts and pancreatic enzymes. An automatic titration unit (902, Metrohm, Herisau, Switzerland) was used to record the volume of 0.1 M NaOH (mL) during intestine digestion (2 h). The FFA release (%) was calculated by the following formula:

(1) $${\%\mathrm{FFA}=100\times (V}_{\mathrm{NaOH}}{\times m}_{\mathrm{NaOH}}{\times M}_{\mathrm{Lipid}}{\;)/(W}_{\mathrm{Lipid}}\times 2)$$

where V_NaOH_ (mL) and m_NaOH_ refer to the volume and molarity of NaOH. W_Lipid_ (g) and M_Lipid_ represent the total weight of the lipid phase, and the molecular weight of the corn oil was about 800 g/mol.

### 2.6. Emulsion Characterization

Droplet size. The particle size ($D_{43}=\Sigma n_{i}{d_{i}}^{4}/\Sigma n_{i}{d_{i}}^{3}$) and distribution were determined by a laser diffraction particle size analyzer (Mastersizer 3000, Malvern instruments Ltd., Malvern, UK). The refractive index values of the oil and aqueous phases were taken to be 1.48 and 1.33, respectively. Five replicates were carried out for each determination.

Zeta potential. A Nano-ZS Laser light scattering instrument was used to measure the zeta potential of the samples (MPT-2, Malvern instruments Ltd., Malvern, UK). All measurements were performed using duplicate samples that were diluted, and each sample was run three times.

Microscopy. The microstructures were observed using a fluorescence confocal laser scanning microscope (LSM710, ZEISS, Germany) with a magnification of 500. The samples (1 mL) were mixed with 20 μL of freshly prepared Nile red solution (1 mg/mL in EtOH) with a 15 s vortex. The stained emulsion was placed on a microscope slide (7101, Sail Brand, China) and then covered by a coverslip (10212020C, Citoglas, China). The emission and excitation wavelengths were set at 623 nm and 488 nm, respectively.

A scanning electron microscope (SEM, Zeiss Sigma VP, German) was applied to determine the LCNF distribution at the interface of the polymerized PS beads [31], even though the analysis was conducted with solid beads from a different organic phase due to constraints in directly obtaining comparable data in the fluid system [32]. The diluted emulsion was dropped onto the holder, followed by drying at ambient temperature. Subsequently, the dried samples were metallized with a platinum/palladium coating and observed under an accelerated voltage of 2 kV.

### 2.7. Statistical Analysis

All experimental data were reported as the means ± standard deviations (SD). Tukey’s test was used to evaluate significant differences (*p* < 0.05), which was conducted in SPSS 16.0 (SPSS Inc., Chicago, IL, USA).

## 3. Results and Discussion

### 3.1. LCNF Properties

#### 3.1.1. Morphology

**Figure 1:**
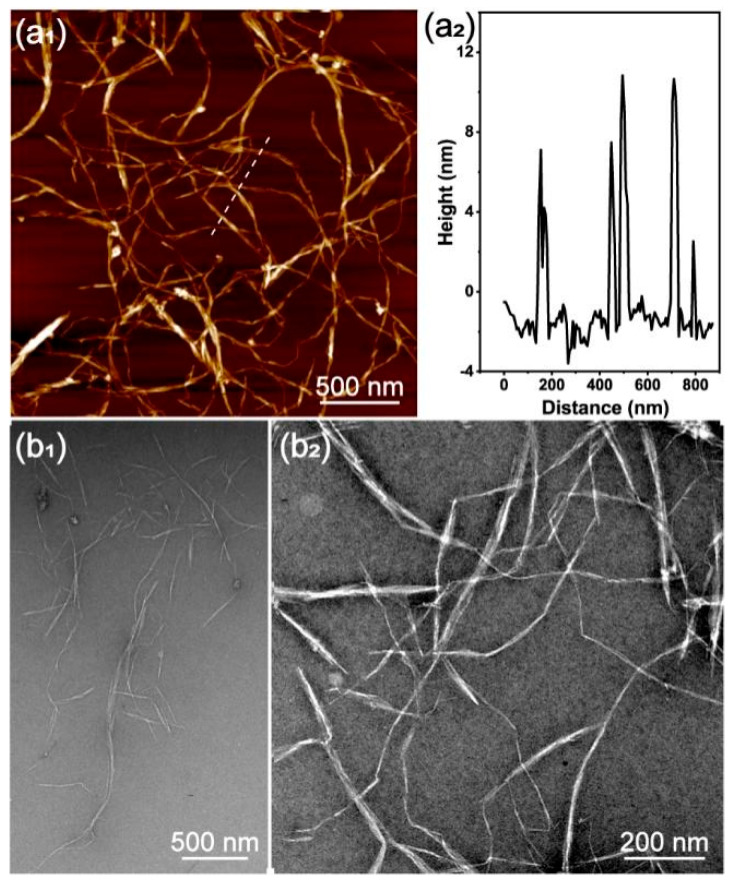
Morphology observation of lignin−containing cellulose nanofibrils (LCNFs): (**a**) atomic force microscope (AFM) image (**a_1_**) with height distribution (**a_2_**); (**b**) transmission electron microscope (TEM) images of LCNFs with different magnification, (**b_1_**) ×5000, (**b_2_**) ×15,000.

The typical morphology and height of LCNFs are shown in Figure 1. The LCNFs displayed a slightly entangled fibril network (Figure 1(a_1_)) with an AFM height dimension (width) ranging from 4 to 12 nm (Figure 1(a_2_)). TEM images of the LCNF microstructure were consistent with the AFM image, in which most of the LCNFs were less than a micron in length. The AFM and TEM images showed that the pulp fibers were converted to individual LCNFs, owing to mechanical homogenization and *p*-TsOH treatment. The constrained resolution of the AFM and TEM images was influenced by the small scan sizes and the complex fibrous network structures, thereby limiting the precision of measurements. Despite these limitations, the derived values were considered acceptable for our research purposes, considering the methods used and the inherent variation in the dimensions of the LCNFs.

#### 3.1.2. Interfacial Tension

**Figure 2:**
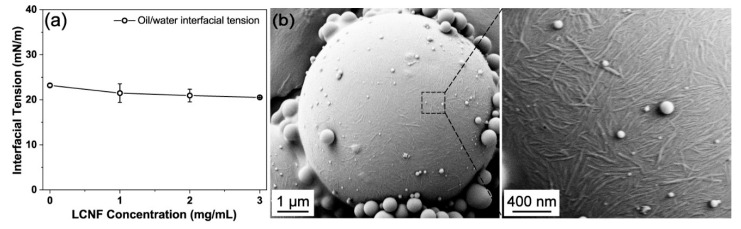
Surface coverage of the lignin-containing cellulose nanofibrils (LCNFs) on polystyrene (PS) beads in the St-in-water Pickering emulsions: (**a**) interfacial tension measured at the core oil–water interface; (**b**) scanning electron microscope (SEM) images of PS beads obtained from the polymerized emulsions that were stabilized by LCNFs.

Interfacial tension was conducted to assess the LCNF capability and elucidate the stabilization mechanism [33]. The effects of varying LCNF concentrations on the interfacial tension are illustrated in Figure 2a. No significant changes in interfacial tension values were observed. Traditional emulsifiers are known to reduce the interfacial tension, promoting droplet breakage during emulsification and enhancing the emulsion’s stability [34]. In contrast, the LCNFs acted differently by forming a steric barrier at the interface through irreversible attachment, rather than through interfacial activity. This Pickering mechanism of LCNFs can effectively prevent coalescence in emulsion systems.

#### 3.1.3. LCNFs at Interfaces in Pickering Emulsions

The limited interfacial activity of LCNFs necessitates further investigation into their potential adsorption at the interface of oil droplets. The LCNF coverage in the droplet surface was analyzed in experiments involving preparation of the LCNF-stabilized St-in-water Pickering emulsion (Figure 2b). The polystyrene beads exhibit a uniform coating of a monolayer film consisting of nanofibrils. This specific interfacial structure contributes to steric hindrance, which played a key role in the effective performance of LCNFs as Pickering stabilizers. Moreover, the presence of small beads adhering to the surface of larger beads can be attributed to outgrowth phenomena during styrene polymerization [35]. The monomer was polymerized with the thermal activation of AIBN initiating the reaction, while the free LCNF complexes served as nucleation sites.

### 3.2. Effect of LCNF Concentrations on Lipid Droplets during Digestion Stages

To assess the influence of LCNF coatings on the digestion behavior of lipid droplets, Pickering emulsions were formulated with varying concentrations of LCNFs and subjected to an *in vitro* gastrointestinal model. In our previous work, we highlighted the importance of considering variations in ionic strength, pH, interfacial composition, and intricate interactions when assessing surface charge, droplet size, and microstructure throughout a three-stage gastrointestinal process [5]. These factors are likely to influence the properties of LCNFs attached to droplet surfaces, thereby complicating measurements during the respective gastrointestinal stage [13].

#### 3.2.1. Evolution of Lipid Droplet Diameter and Distribution

**Figure 3:**
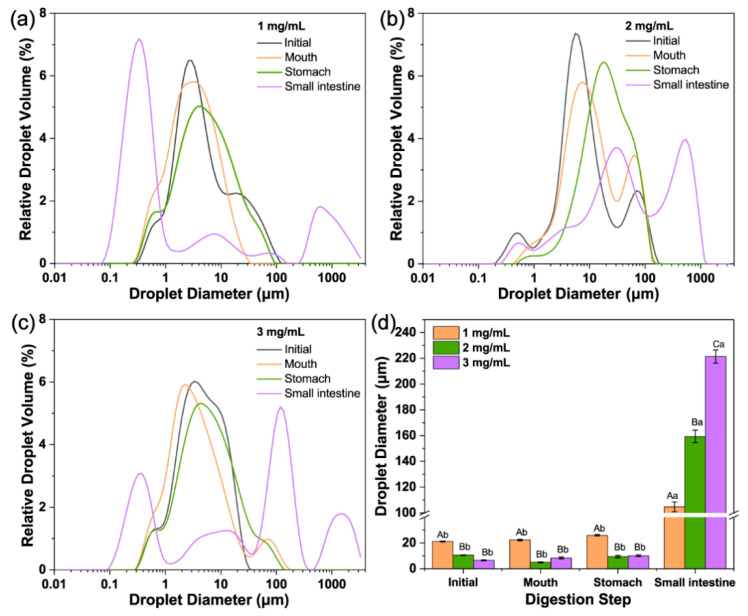
Size distribution of corn-oil-in-water Pickering emulsions stabilized by (**a**) 1 mg/mL, (**b**) 2 mg/mL, and (**c**) 3 mg/mL lignin-containing cellulose nanofibrils (LCNFs) and (**d**) the mean droplet diameter (*D*_43_) changes at different gastrointestinal stages. The different capital letters (A to C) indicate significant differences (*p* < 0.05) within the same digestion stages. The different lowercase letters (a, b) indicate significant differences (*p* < 0.05) during different digestion stages.

**Figure 4:**
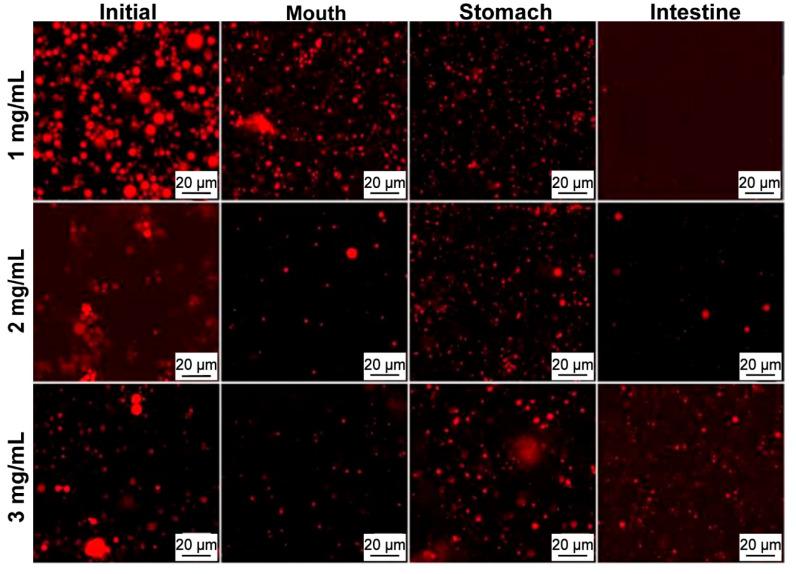
CLSM images of lignin-containing cellulose nanofibril (LCNF)-stabilized Pickering emulsions at different gastrointestinal tract stages.

The droplet sizes and distribution of LCNF-stabilized Pickering emulsions after digestion are illustrated in Figure 3. Three emulsions with varying LCNF loadings of 1, 2, and 3 mg/mL were prepared, each with mean diameters of 21.1 µm, 10.5 µm, and 6.5 µm, respectively. The LCNF loading significantly influenced the mean droplet diameter (*p* < 0.05). The droplet distribution of the 1 and 2 mg/mL LCNF emulsions exhibited less uniformity (multimodal), whereas the droplet size distribution was monomodal at 3 mg/mL LCNFs. Notably, there was a presence of peaks smaller than 1 µm in the three emulsions owing to the dispersion of LCNFs in the continuous phase. At lower concentrations, the presence of LCNFs in the emulsion system was insufficient to effectively stabilize the droplets formed during sonication. As a result, the droplets experienced rapid coalescence. In contrast, emulsions prepared with 3 mg/mL of LCNFs as the Pickering stabilizer exhibited enhanced surface accessibility, leading to increased stabilization of the droplets and ultimately resulting in smaller droplet sizes with the increased interfacial area. Furthermore, there was a phenomenon of minor droplet aggregations observed in all emulsions, as depicted in the confocal laser scanning microscope (CLSM) images (Figure 4). This can be explained by the inherent tendency to entangle, causing bigger-sized droplets, which promoted coalescence [18]. Another potential reason could be the dispersion of non-adsorbed LCNFs in the continuous phase, leading to flocculation and affecting droplet morphology [36].

Following exposure to the simulated mouth, the peak positions in the size distribution curves remained consistent with those of the original emulsions (Figure 3 and Figure 4), indicating the relative stability of LCNF-coated droplets under oral conditions. Moreover, no significant changes in the diameter values (*p* > 0.05) were observed for the three samples after mouth digestion. The stability of these droplets suggested that LCNF-stabilized Pickering emulsions may exhibit resistance to mucin from simulated saliva. It is commonly understood that the physical barrier from particles offers enhanced stability.

The average droplet sizes of the three LCNF-stabilized emulsions after gastric digestion exhibited slight fluctuations (*p* < 0.05). These droplets remained relatively small and showed no significant differences among every emulsion (*p* > 0.05), which was confirmed by a CLSM (Figure 4). The spherical droplet structure was sustained under highly acidic gastric conditions, indicating that the LCNF coatings effectively protected against droplet aggregation. Moreover, the behavior of LCNF-coated lipid droplets differed from that of protein-stabilized droplets (sodium caseinate), which displayed a tendency to flocculate [37,38].

After small intestinal digestion, significant increases in mean droplet diameter were observed for all three types of emulsions (*p* < 0.05), accompanied by noticeable changes in microstructures, as illustrated in Figure 3 and Figure 4. The droplet diameters were larger than 100 µm after digestion as a result of the hydrolysis by pancreatic lipase. Interestingly, the peak of the particle size distribution exhibited significant variations, possibly due to several factors. The digestion process might have led to the removal of some of the oil phase from the emulsion, leading to a higher volume fraction of LCNFs in the particle size distribution [39]. This shift was particularly noticeable in the case of the 1 mg/mL LCNF-stabilized emulsion, as shown in Figure 3a, where a distinct peak below 1 µm was observed after the intestinal digestion. Furthermore, the interaction of various components (e.g., free fatty acids and bile acids) might have contributed to the aggregation or flocculation of the emulsions. This process may have posed challenges in observing individual emulsion droplets [40], further supporting the earlier hypothesis regarding the changes in particle size distribution after digestion.

#### 3.2.2. The Zeta Potential of Emulsions

**Figure 5:**
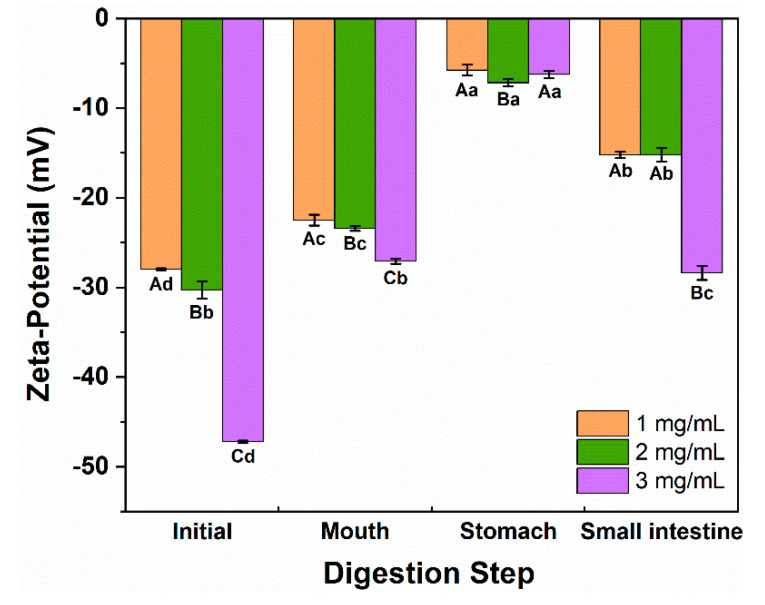
Zeta potential of LCNF-stabilized Pickering emulsions at different gastrointestinal tract stages. Emulsions were stabilized by different levels of LCNFs (as noted). The different capital letters (A to C) indicate significant differences (*p* < 0.05) within the same digestion stages. The different lowercase letters (a to d) indicate significant differences (*p* < 0.05) during different digestion stages.

The emulsion interface composition was evaluated during the GIT digestion process, and the impact of LCNFs on triglyceride (TAG) hydrolysis was further analyzed by monitoring changes in charge (Figure 5). All emulsions exhibited a negative charge, and the evolution trend was similar. The strength was varied based on the LCNF loading at the interface. The zeta potentials of the initial emulsions stabilized with 1, 2, and 3 mg/mL of LCNFs were −28.0, −30.3, and −47.2 mV, respectively, displaying a significant difference (*p* < 0.05). The zetapotential absolute values of all emulsions significantly decreased as they were transferred from the initial to the mouth phase (*p* < 0.05). This can be attributed to electrostatic screening of the surface potential [41].

A further decrease in absolute values was observed after gastric digestion owing to the low pH and a high ionic environment. This transition also influenced the electrostatic interactions of emulsions. Specifically, the cationic proteins in gastric fluids could reduce the surface potential [13].

In comparison with the gastric phase, a stronger negative charge was found in all samples (*p* < 0.05) after the intestinal digestion owing to the displacement of various anionic substances like FFA and bile acids [42]. Overall, the emulsions exhibited the highest absolute values in the initial phase but displayed the lowest values after the stomach phase. Similar results have been reported previously, where the digestion properties of Pickering emulsions stabilized by nanoparticles exclusively featured either negative or positive charge groups, leading to more significant fluctuations in the zeta potential [9].

#### 3.2.3. Free Fatty Acid (FFA) Release

**Figure 6:**
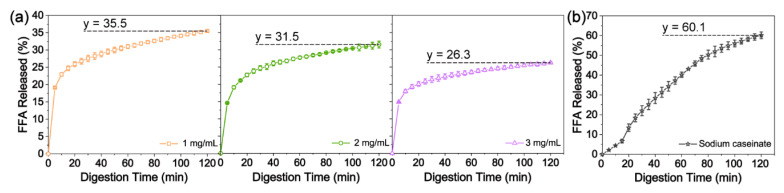
Release of FFAs during simulated small intestinal digestion of LCNF-stabilized corn-O–W Pickering emulsions (**a**) and sodium caseinate-stabilized O–W emulsion (**b**).

The impact of Pickering stabilizer concentration on the lipid digestion profile was evaluated, and the findings are depicted in Figure 6. A consistent trend in the FFA release was observed. FFA release sharply increased within the initial 15 min and then plateaued at around 120 min. This pattern corroborates with previous research studies [5]. Nonetheless, variations in the digestion profiles were observed based on the emulsifier concentration. The highest degree of lipid digestion (35.5%) was observed in emulsions coated with 1 mg/mL of LCNFs, while the lowest FFA released value (26.3%) was recorded for emulsions with 3 mg/mL of LCNFs. This discrepancy can be explained by the better accessibility of lipid droplet surfaces with lower LCNF loading to lipase digestion as compared to those with higher LCNF concentrations. Sodium caseinate derived from bovine milk was used here as a protein emulsifier and is commonly used in the food industry [43]. After 120 min of digestion, the LCNF-stabilized Pickering emulsions showed a significant reduction in lipid hydrolysis compared to the sodium caseinate-stabilized emulsion with 60.1% of FFA release.

The droplets stabilized by a higher dose of LCNFs exhibited a larger initial specific surface area, potentially increasing the exposure of the lipid surface to intestinal lipase and thus promoting enhanced digestion. The experimental results, on the contrary, show that the emulsion system with the smallest droplets showed the lowest degree of digestion, with a gradual FFA increase from approximately 21.4% at 30 min to 26.3% at 120 min (Figure 3d and Figure 6). Several physicochemical phenomena were likely to contribute to the lower lipid digestion in the emulsion with a higher LCNF content. The irreversible adsorption of LCNFs onto the interface could inhibit the binding of lipases and bile salts. Additionally, the LCNFs could form a dense physical barrier, restricting the lipase’s access to the lipids at higher levels. This can be supported by SEM displaying a thick layer of LCNFs surrounding the PS beads (Figure 2b). The free LCNFs in the continuous phase may lead to the formation of a network of aggregated fibers, increasing viscosity and impeding the movement of lipase molecules [4]. Furthermore, negatively charged LCNFs have the potential to bind free calcium ions, thus affecting their effectiveness in removing FFAs from the interface and inhibiting subsequent digestion [10]. Consequently, the LCNF-induced delayed lipid hydrolysis observed can be attributed to the formation of a less penetrable coating and network that encircle the oil droplets, providing valuable insights into the underlying mechanism of lipid digestion delay with LCNFs.

## 4. Conclusions

In this study, we prepared cellulose nanofibrils containing lignin (LCNFs) and used them to stabilize corn-oil-in-water Pickering emulsions. We also investigated their impacts on lipid digestion behaviors. The results indicated that the lipid hydrolysis extent was significantly affected by the concentrations of LCNFs. Higher levels of LCNFs led to a reduction in the lipolysis degree. This can be explained by the formation of a physical barrier on the interface and the creation of a network structure between them. The FFA released amounts were 35.1%, 31.5%, and 26.3% for LCNF loadings of 1 mg/mL, 2 mg/mL, and 3 mg/mL, respectively. The relatively low FFA values suggested that the adsorbed LCNF layer exhibited some permeability to lipase molecules, allowing them to reach the emulsified oil. Overall, the LCNFs demonstrated the ability to inhibit lipase activity by forming Pickering emulsions and subsequently forming flocculates that act as a protective barrier, shielding the lipid from interacting with and being hydrolyzed by lipase. This potential mechanism offers a promising strategy (e.g., emulsion system and oil powder) for reducing fat absorption from food and assisting in weight loss.

## Data Availability

The data presented in this study are available on request from the corresponding author.
